# Expanded Chinese hamster organ and cell line proteomics profiling reveals tissue-specific functionalities

**DOI:** 10.1038/s41598-020-72959-8

**Published:** 2020-09-28

**Authors:** Kelley Heffner, Deniz Baycin Hizal, Natalia I. Majewska, Swetha Kumar, Venkata Gayatri Dhara, Jie Zhu, Michael Bowen, Diane Hatton, George Yerganian, Athena Yerganian, Robert O’Meally, Robert Cole, Michael Betenbaugh

**Affiliations:** 1 Department of Chemical and Biomolecular Engineering, Johns Hopkins University, Baltimore, MD USA; 2 AstraZeneca, Cell Culture and Fermentation Sciences, Gaithersburg, MD USA; 3 Allogene Therapeutics, Product and Process Development, South San Francisco, CA USA; 4 Cytogen Research and Development, Inc, Boston, MA USA; 5 Department of Pathology, Johns Hopkins School of Medicine, Baltimore, MD USA

**Keywords:** Biologics, Proteome informatics

## Abstract

Chinese hamster ovary (CHO) cells are the predominant production vehicle for biotherapeutics. Quantitative proteomics data were obtained from two CHO cell lines (CHO-S and CHO DG44) and compared with seven Chinese hamster (*Cricetulus griseus*) tissues (brain, heart, kidney, liver, lung, ovary and spleen) by tandem mass tag (TMT) labeling followed by mass spectrometry, providing a comprehensive hamster tissue and cell line proteomics atlas. Of the 8470 unique proteins identified, high similarity was observed between CHO-S and CHO DG44 and included increases in proteins involved in DNA replication, cell cycle, RNA processing, and chromosome processing. Alternatively, gene ontology and pathway analysis in tissues indicated increased protein intensities related to important tissue functionalities. Proteins enriched in the brain included those involved in acidic amino acid metabolism, Golgi apparatus, and ion and phospholipid transport. The lung showed enrichment in proteins involved in BCAA catabolism, ROS metabolism, vesicle trafficking, and lipid synthesis while the ovary exhibited enrichments in extracellular matrix and adhesion proteins. The heart proteome included vasoconstriction, complement activation, and lipoprotein metabolism enrichments. These detailed comparisons of CHO cell lines and hamster tissues will enhance understanding of the relationship between proteins and tissue function and pinpoint potential pathways of biotechnological relevance for future cell engineering.

## Introduction

The history of CHO cell line establishment from *Cricetulus griseus* dates back to the 1950s when Theodore T. Puck and colleagues established the CHO-K1 cell line^1^. Today, CHO cell lines generated from the Chinese hamster dominate the biopharmaceutical industry and their products generate billions of dollars in revenue annually^2,3^. The success of CHO cell lines for recombinant protein production can be attributed to their high growth rate, ease of genetic modification, and ability to post-translationally modify proteins via glycosylation, including galactosylation and sialylation. The original CHO cell line was adapted and modified by various researchers to create cell lines, such as CHO-DXB11, CHO DG44, and CHO-S^4,5^. Thus, there may be genetic differences across CHO parental cell lines, as well as clonal- and process- dependent variations^2^. These clonal variabilities may potentially lead to differences across transcriptomes and proteomes. Since each and every CHO cell line exhibits significant genomic, transcriptomic, and proteomic signatures, a single CHO-ome is not necessarily directly applicable across different laboratories. In addition to cell line differences, variations in the bioprocess conditions, including media formulations and bioreactor operations, can alter the transcriptome and proteome.

Initial efforts to understand CHO include the sequencing of both the CHO and *Cricetulus griseus* genomes. The draft CHO-K1 genome was established in 2011^6^, and the Chinese hamster genome followed two years later^7^. In addition, the CHO-DXB11 genome was sequenced to understand the DHFR negative phenotype and cell line drift^8^. Other efforts have focused on creating bacterial artificial chromosome libraries for CHO-K1 and CHO-DG44 cell lines in order to visualize hamster chromosome re-arrangements^9^. The recent PICR Chinese hamster genome has utilized real time sequencing with Illumina-based assemblies to generate a more complete CHO genome^10^.

Similar efforts have been used to understand mRNA and protein expression in CHO using transcriptomics and proteomics, respectively. Through advancements in sample preparation and mass spectrometry (MS) technology, it is possible to identify and quantify thousands of cellular proteins. Initial CHO proteomic analyses revealed enrichment in protein processing and apoptosis pathways at the proteomic level in the CHO-K1 cell line compared to the human, mouse, and CHO genome and transcriptome^11^. More recent studies in our lab and others have revealed extensive CHO proteomics databases as well as databases for the secretome and CHO cell lines in exponential and stationary phases^12,13^. In addition, different bioprocess conditions have been studied via transcriptomics and proteomics to yield insights into protein production, cell growth, cell death, favorable glycosylation, and optimized media formulations^14,15^.

Proteomics has been used to study the proteome of specific organs in order to elucidate how protein expression changes across different tissues in the hamster, as previously studied for human^16^ and mouse^17^. Hamsters represent another important small animal model in biomedicine for studying diseases and evaluating the effect of potential therapies in pharmaceutical sciences. Indeed, Chinese hamsters have been applied as a small animal model for diabetes, cancer, and the impact of radiation^18–21^. More recently, hamster species represent one of the prime small animal models for examining the impact of SARS-CoV-2 infections and potential therapies^22^. Understanding the proteomics profiles across different tissues will help biomedical scientists to understand how expressed proteins are responsible for tissue-specific functionalities. From a disease perspective, tissue proteomics can enable scientists to appreciate why different diseases and drugs affect various tissues differently. From a biotechnology perspective, specific functionalities present in different tissues can provide insights into ways to improve the capabilities of CHO cell line production hosts by adding capabilities present in certain tissues that may be distinct from current hosts.

Therefore, in this study, we employed comparative proteomics using tandem mass tag labeling (TMT) in order to compare two representative CHO cell lines and seven different tissues from Chinese hamster. The CHO-S and CHO DG44 cell lines were used as model cell lines for comparison to hamster tissue expression patterns. Multiple organs (brain, heart, kidney, liver, lung, ovary and spleen) relevant to pharmaceutical sciences, disease, and biotechnology were used to generate tissue-specific proteomes, providing the most comprehensive and diverse tissue proteome available for hamsters to date. Tissue-tissue and tissue-cell line comparisons suggest functions and pathways with significant differential expression across different cell types, with cell lines tending to upregulate proteins associated with growth and gene expression, while tissue samples exhibit upregulation in tissue-specific functional pathways. Examining proteins in CHO cell lines and tissues will enhance our understanding of why tissue exhibit certain characteristics, how cell lines are adapted for cell culture and protein production, and highlight how CHO cells could potentially be modified to include useful tissue-specific functions in future cell engineering efforts.

## Materials and methods

### Tissue isolation

Tissues from various organs (brain, heart, kidney, liver, lung, ovary and spleen) were harvested from female Chinese hamsters (age 5–10 weeks) generously provided by the lab of Dr. George Yerganian (Cytogen Research and Development, Boston, MA, USA). Euthanization was performed by CO_2_ and verified by abdominal puncture, in accordance with all guidelines and regulations of the Johns Hopkins University Animal Care and Use Committee (Approved Protocol HA14A84). Upon harvest, each organ was divided into small pieces, rapidly frozen on dry ice, and subsequently stored at − 80 °C until further analysis.

### Cell culture

Two commercial suspension CHO cell lines, CHO-S and CHO DG44, were grown in batch culture and the samples were collected and stored. CHO-S cells were cultured in CD-CHO medium supplemented with 8 mM glutamine (ThermoFisher Scientific, Waltham, MA, USA), and CHO DG44 cells were cultured in DG44 medium supplemented with 2 mM glutamine (ThermoFisher Scientific, Waltham, MA, USA). Both cell lines were incubated at 37 °C with 8% CO_2_ shaking at 120RPM; viable cell density and viability were determined by hemocytometer and trypan blue staining. For sample collection, approximately 3 × 10^6^ cells were centrifuged, washed with PBS on ice, frozen rapidly on dry ice and stored at −80 °C until analysis. Based on the growth profiles for both cell lines (data not shown), samples collected on day 2 were identified as corresponding to mid-exponential phase and used for further analyses.

### Proteomics sample preparation

Samples for proteomics were thawed on ice and lysed in a solution of 2% sodium dodecyl sulfate (SDS) in 500 µL of cell lysis buffer supplemented with 0.1 mM phenylmethylsulfonyl fluoride (PMSF) and 1 mM ethylenediaminetetraacetic acid (EDTA), pH 7–8. Lysates were sonicated x3 times for 60 s at 20% amplitude followed by a 90 s pause. Protein concentration was measured by a bicinchoninic acid (BCA) protein assay. One hundred micrograms of each sample were reduced in 10 mM tris(2-carboxyethyl) phosphine (TCEP), pH 7–8, at 60 °C for 1 h on a shaking platform. Iodoacetamide was added to alkylate the sample to a final concentration of approximately 17 mM and incubated for 30 min in the dark. Next, samples were passed through 10 kDa filters to reduce the SDS concentration as described in the filter-aided sample preparation (FASP) method^23^. During the FASP method, tetra-butyl ammonium bicarbonate (TEABC) was added after the urea washes to increase protein recoverability from the filters. The samples were finally digested using trypsin/LysC enzyme mix (Promega V507A) at an enzyme to substrate ratio of 1:10, overnight at 37 °C on a shaking platform. After digestion, peptides were cleaned up by C18 cartridges and labeled with TMT reagents. All TMT labeled samples were combined and vacuum centrifuged to dryness removing the entire liquid.

### Labeling

In order to compare protein expression, samples were labeled in duplicate (biological replicates) using two TMT-10plex labeling kits (ThermoFisher Scientific, Waltham, MA, USA). Triplicates were used for ovary tissue and CHO-S. Each of the 10 reagents has the same nominal mass and chemical structure, so that for each sample a unique reporter mass (126–131 Da) was used to relate protein expression levels. Specifically, we included technical and biological replicates of CHO-S (two samples in TMT 1 and one sample in TMT 2) to aid in comparisons between and within the TMT experiments. All other samples contained only biological replicates, which were randomly assigned to one of the TMT-10plex kits. The TMT labeling design is provided in Table 1. Following protein digestion, TMT reagents were thawed, and acetonitrile was used to dissolve the reagents. One reagent tube was added to each sample and then incubated at room temperature for 1 h. Hydroxylamine was subsequently added to quench the reaction before the tubes were combined. All TMT labeled samples were combined and vacuum centrifuged to dryness, removing the entire liquid. Each TMT-10plex was subjected to analysis by two-dimensional liquid chromatography tandem MS (2D LC/MS/MS).

**Table 1:** TMT experimental design.

TMT 1		TMT 2	
Tag	Sample	Tag	Sample
127_C	Brain replicate 1	127_C	Liver replicate 1
127_N	Lung replicate 1	127_N	Heart replicate 1
128_C	Kidney replicate 1	128_C	Ovary replicate 1
128_N	Brain replicate 2	128_N	Liver replicate 2
129_C	Spleen replicate 1	129_C	CHO DG44 replicate 1
129_N	Kidney replicate 2	129_N	Ovary replicate 2
130_C	CHO-S replicate 1	130_C	Ovary replicate 3
130_N	Spleen replicate 2	130_N	CHO DG44 replicate 2
126	Lung replicate 2	126	Heart replicate 2
131	CHO-S technical replicate	131	CHO-S technical replicate

### 2D LC–MS/MS

Digested peptides were fractionated using an Agilent 1200 Capillary LC with a 254 nm Agilent variable wavelength detector on a basic reversed phase column (XBridge C18 Guard Column, 5 µm, 2.1 × 10 mm XBridge C18 Column, 5 µm, 2.1 × 100 mm) at a flow rate of 250 µL/min. The gradient was set from 100% A to 100% B (A = 10 mM Triethyl Ammonium Bicarbonate (TEAB) and B = 90% acetonitrile, 10 mM TEAB). 84 fractions were collected over 105 min and concatenated into final 24 samples prior to second dimension LC and MS analysis. Tandem mass spectrometry analysis of the peptides was carried out on the LTQ-Orbitrap Velos (ThermoFischer Scientific, Waltham, MA, USA) MS interfaced to Eksigent (Eksigent, Dublin, CA, USA) nanoflow liquid chromatography system with an Agilent 1100 autosampler (Agilent, Santa Clara, CA, USA). Peptides were enriched on a 2 cm trap column (YMC Americas, Allentown, PA, USA), fractionated on Magic C18 AQ, 5 µm, 100 Å, 75 µm × 15 cm column (Michrom Bioresources, Auburn, CA, USA) and electrosprayed through a 15 µm emitter (New Objective, Woburn, MA, USA). The reversed-phase solvent gradient consisted of solvent A (0.1% formic acid) with increasing levels of solvent B (0.1% formic acid, 90% acetonitrile) over a period of 90 min. LTQ Orbitrap Velos parameters included 2.0 kV spray voltage, full MS survey scan range of 350–1800 *m/z*, data dependent HCD MS/MS analysis of top 10 precursors with minimum signal of 2000, isolation width of 1.9, 30 s dynamic exclusion limit and normalized collision energy of 35. Precursor and the fragment ions were analyzed at 60,000 and 7500 resolutions, respectively.

### Bioinformatics

Peptide sequences were identified from isotopically resolved masses in MS and MS/MS spectra extracted with and without deconvolution using Thermo Scientific MS2 processor and Xtract software nodes. Data were searched against all entries in the *Cricetulus griseus* database (Proteome Discoverer v1.4) for CHO cell lines. Oxidation on methionine, deamidation NQ, and phosphoSTY were set as variable modifications, and carbamidomethyl on cysteine was set as fixed modifications in Mascot search node used in Proteome Discoverer (ThermoFischer Scientific, Waltham, MA, USA) workflow. Mass tolerances on precursor and fragment masses were 15 ppm and 0.03 Da, respectively.

Protein identifications were made using Proteome Discoverer software with a high confidence cutoff [< 1% false discovery rate (FDR)]. The protein intensities were evaluated by fold change, using the CHO-S cell line technical replicate in each TMT as the basis. Protein accession numbers were mapped to gene symbols using the biological database network (https://biodbnet-abcc.ncicrf.gov) for functional analysis by gene ontology (GO). For GO annotation, gene symbols were mapped to biological processes, using the GO-CHO platform (https://ebdrup.biosustain.dtu.dk/gocho). All programming for the hypergeometric test were calculated in MATLAB version 2015vB (https://www.mathworks.com/products/matlab) and RStudio (https://www.rstudio.com). Enrichment and depletion p-values were calculated using the hygecdf and hygepdf functions in MATLAB. These values were used to generate heat maps and k-means clustering in Genesis software version 1.7.6^24^. Pathways of interest were plotted and evaluated using IPA software (https://www.ingenuity.com/).

## Results and discussion

**Figure 1:**
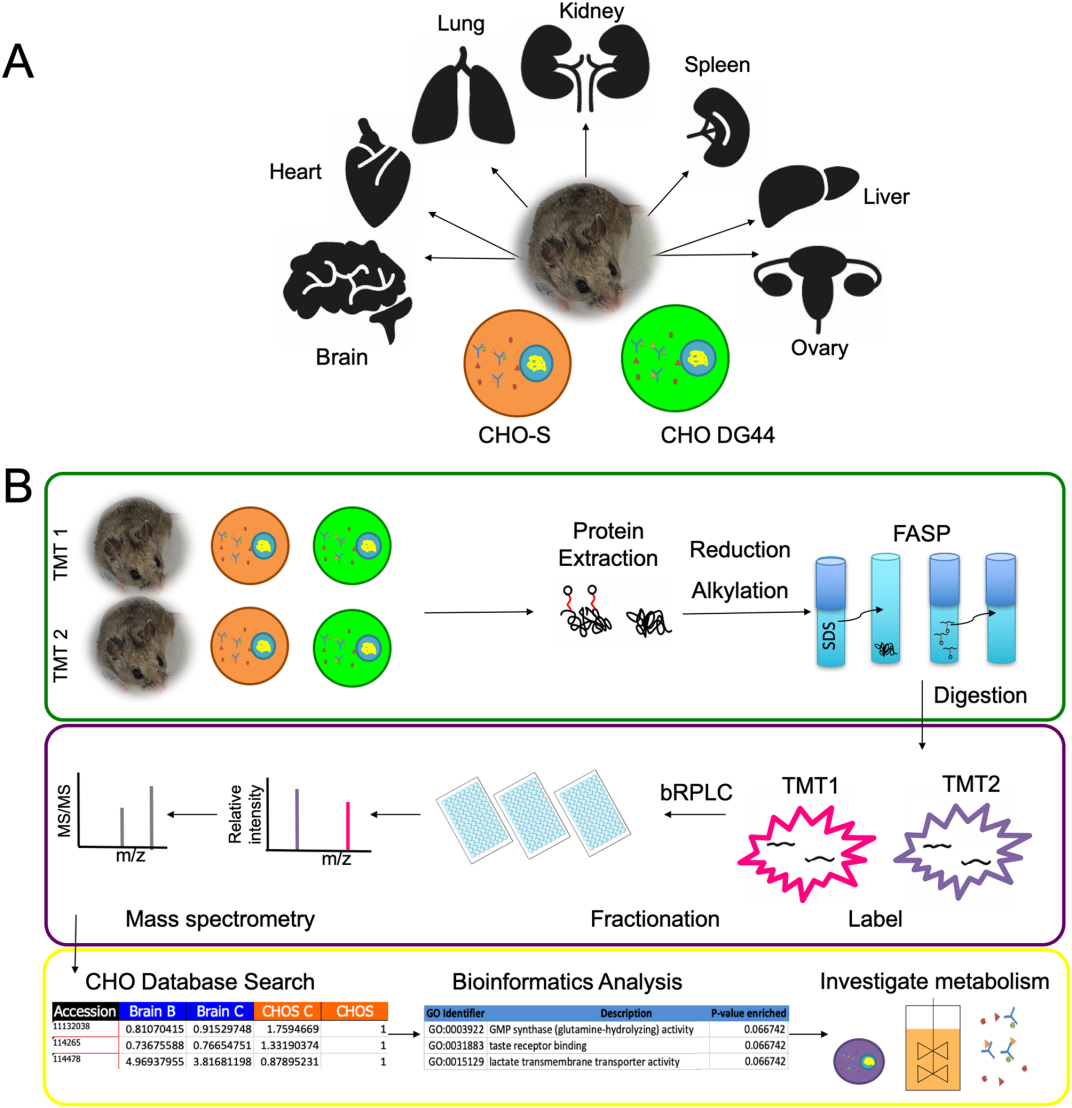
Overview of experimental design and workflow. (**A**) Schematic of the proteome experiment comparing hamster tissues (brain, heart, lung, kidney, spleen, liver and ovary) and CHO cell lines (CHO-S and CHO DG44). (**B**) Each TMT 10-plex contained a mix of tissues and cell line samples. Sample preparation involved protein extraction from tissues and cells, followed by reduction, alkylation, FASP, and digestion. Peptides were labeled and combined into two TMT 10-plex experiments for fractionation and mass spectrometry identification. In the bioinformatics analysis pipeline, peptides were matched to proteins and gene symbols for functional analysis in order to understand cell and tissue metabolism. *TMT* tandem mass tag, *FASP* filter aided sample preparation, *bRPLC* basic reversed phase liquid chromatography.

This study was undertaken to compare protein expression of various CHO cell lines and hamster tissues, resulting in the most comprehensive multi-tissue analysis of the *Cricetulus griseus* proteome (Fig. 1A). This multi-tissue and multi-cell line analysis aims to improve our understanding of the Chinese hamster as the original tissue source for CHO cell lines. Additionally, since CHO is the dominant biopharmaceutical production host in biotechnology, this comparison elucidates similarities and differences across cells and tissues. An overview of the proteomics workflow is shown in Fig. 1B.

Following MS identification, the protein accession numbers were determined using the annotated Chinese hamster genome^25^. Protein accession numbers were converted to gene symbols for functional analysis^26^. For missing gene symbols, the accession numbers were searched against mouse and human databases using the online database, bioDBnet^26^. In this study, gene ontology (GO) and ingenuity pathway analysis (IPA) were used for functional analyses of the differentially expressed proteins. GO analysis converts gene symbols to their molecular function, cellular component, and biological processes in order to evaluate the relationship between the molecular activities of gene products, location of activity, and pathways comprising the activity of multiple gene products, respectively^27^. In another functional analysis, each gene symbol was mapped to the relevant IPA pathways^28^, suggesting enriched and depleted pathways for comparisons between different tissues and cells. For both GO and IPA, the enrichment and depletion values were determined based on p-values that were calculated via the hypergeometric distribution, with p < 0.05 set for evaluating significance.

### Protein identification and total protein intensity differences

#### PCA comparison

**Figure 2:**
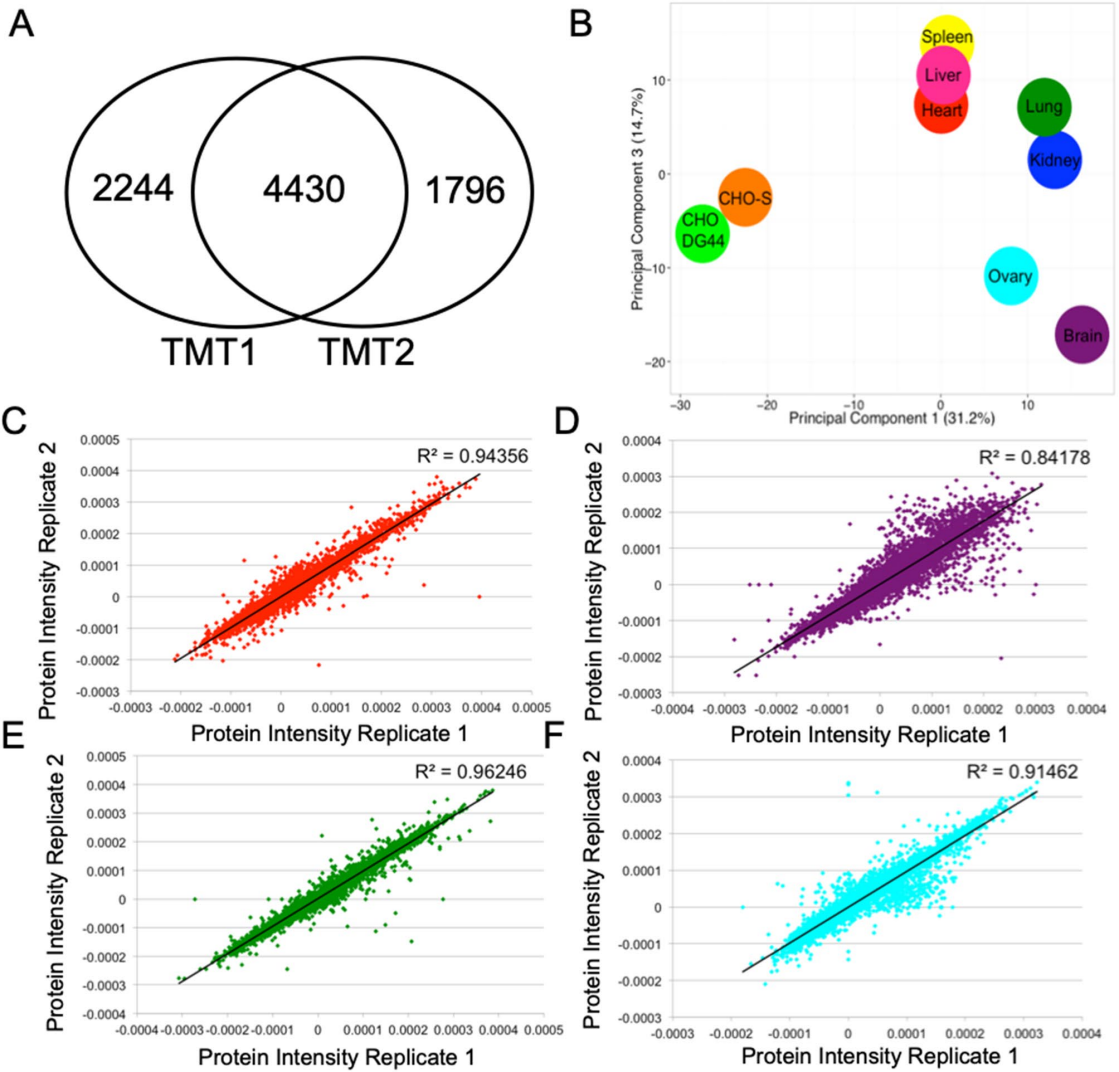
Protein intensity analysis. (**A**) Venn diagram of total proteins identified in each TMT proteomics experiment. (**B**) Principal component analysis of Chinese hamster ovary (CHO) cells and hamster tissues. Clustering of overall protein intensity fold changes was calculated using the first and third principal components to show variation. (**C**–**F**) Normalized protein intensity is plotted for replicates of each tissue corresponding to a different cluster obtained from the principal component analysis. (**C**) Heart, (**D**) brain, (**E**) lung, (**F**) ovary. *TMT* tandem mass tag.

For each sample, the number of unique proteins and peptides identified are listed in Table 2 along with the number of spectra obtained. The complete list of identified proteins and fold change ratios are listed in Supplementary Table 1 and are searchable online through the NIST database (https://peptide.nist.gov). Over 6000 proteins were identified in each sample with at least two unique peptides per protein; this corresponded to a total of 8470 unique proteins containing a false discovery rate (FDR) of 1% for protein identification when the TMT samples were combined and the duplicates were removed (Table 2). Between the two TMT experiments, there were 4430 common proteins identified in both sets (Fig. 2A). A total of 2244 proteins were unique to TMT1 and 1796 proteins were unique to TMT2 (Fig. 2A).

**Table 2:** TMT experiment identification.

Experiment		Unique proteins (#)	Unique peptides (#)	Unique spectra (#)
TMT 1	CHO-S, brain, lung, kidney, brain, spleen, kidney, CHO-S, spleen, lung	6674	51,258	592,903
TMT 2	CHO-S, liver, heart, ovary, liver, CHO DG44, ovary, ovary, CHO DG44, heart	6226	49,860	619,246
		8470 total unique proteins

Protein intensity fold change ratios were initially evaluated through principal component analysis (PCA) of proteins identified in all samples, as shown in Fig. 2B^29^. The PCA distribution represents differences in protein expression for those proteins identified in all samples and is influenced by proteins highly expressed in one tissue versus another. Not surprisingly, as shown by PCA, the cell samples clustered together and were distinct from all the tissues. For the tissue samples, the expression of proteins in spleen, liver, and heart clustered together. Similarly, protein expression in the lung and kidney clustered together. Interestingly, the first cluster (spleen, liver and heart) comprises tissues with dense connective tissue and capillary systems^30^. In contrast, the second cluster (lung and kidney) is specialized for transport, with tubular systems and thin insterstitium^30–33^. Ovary and brain were clustered separately from the other organs.

Next, the two replicates from the same tissue were plotted to examine their consistency. Fitting a curve to the scatter plot and calculating the R^2^ value assessed the linearity of the two replicates. Shown in Fig. 2C–F are the plots for the tissue replicates with the highest R^2^ value for each cluster, specifically lung (from the lung/kidney cluster) and heart (from the heart/kidney/spleen cluster), plus brain and ovary. For the following analyses, we studied these representatives from each of the clusters; additional data on all proteins is provided in Supplementary Table 1.

#### Protein intensity comparisons

**Figure 3:**
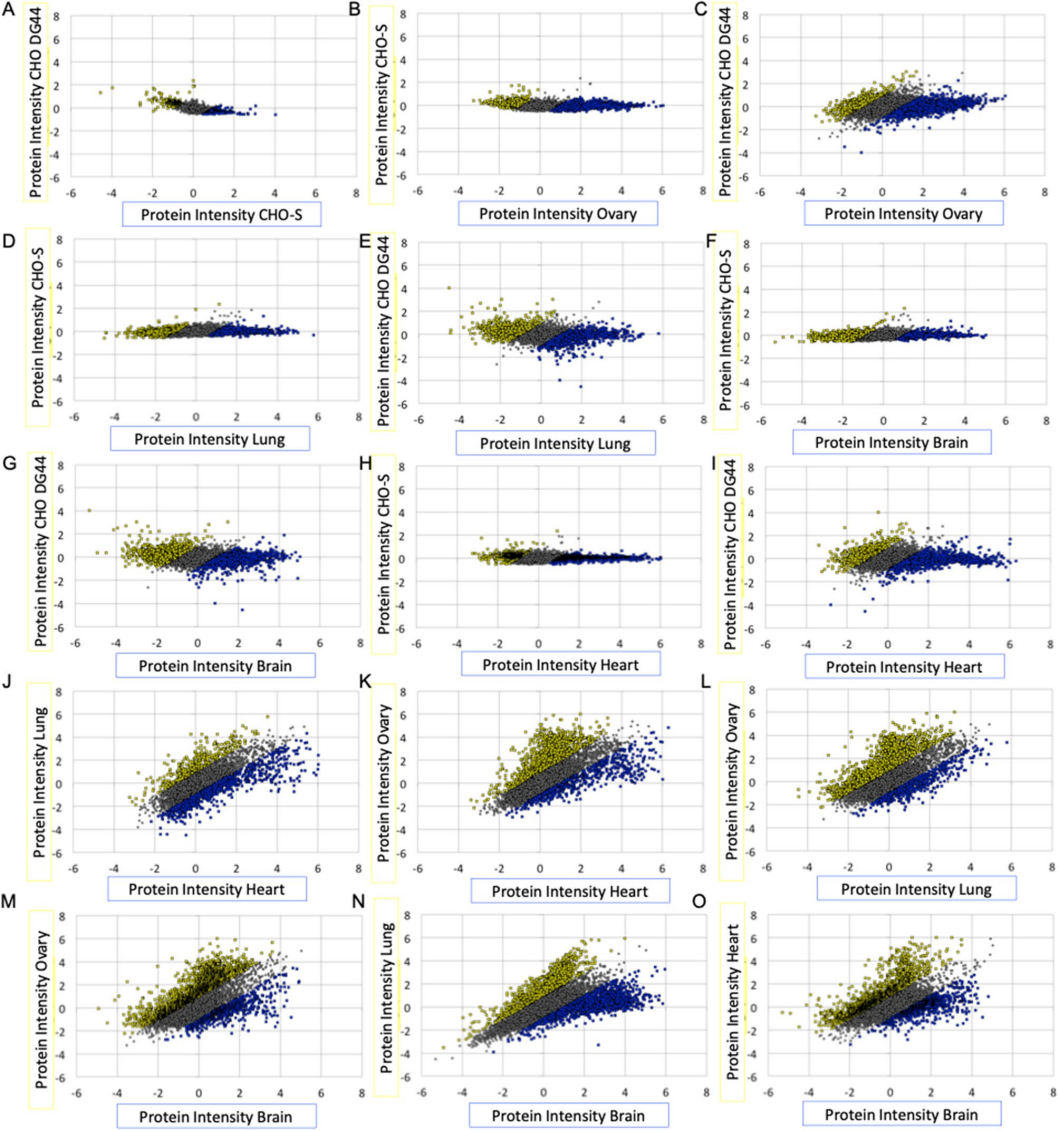
Overall distributions of protein intensity in hamster cells and tissues. Protein intensity was plotted as the average fold change ratio of biological replicates. Yellow represents proteins highly expressed for the sample on the y-axis, while blue represents those on the x-axis.

To compare between cell lines and tissues, protein intensity was averaged between replicates and plotted in Fig. 3. The data was log2 transformed in order to ensure a normal distribution for each sample. A student’s t-test was performed to determine the likelihood of significant differences between samples from cells and tissues. A summary of the statistical analysis, performed using student’s t-test as a means comparison, is shown in Table 3.

**Table 3:** Means comparison using Student’s t-test for total percentage of outliers.

p-value	CHO-S	CHO DG44	Brain	Heart	Lung	Ovary
CHO-S	n/a	0.067	< 0.01*	< 0.01*	< 0.01*	< 0.01*
CHO DG44	0.067	n/a	< 0.01*	< 0.01*	< 0.01*	< 0.01*
Brain	< 0.01*	< 0.01*	n/a	< 0.01*	0.10	0.12
Heart	< 0.01*	< 0.01*	< 0.01*	n/a	0.11	0.051
Lung	< 0.01*	< 0.01*	0.10	0.11	n/a	0.79
Ovary	< 0.01*	< 0.01*	0.12	0.051	0.79	n/a

*p-value < 0.05 indicates that the outlier comparison is statistically significant.

When comparing the cell lines to each other, no significant differences were observed between the CHO-S and CHO DG44 cell lines (Table 2 and Fig. 3A) at a p-value of 0.05. We identified 178 proteins with significantly higher expression in CHO-S relative to CHO-DG44 and 155 proteins with significantly higher expression in CHO DG44 relative to CHO-S, representing the lowest number of total outliers for any comparison. A comparison between cells and tissues is shown in Fig. 3B through I. For these comparisons, the total number of proteins with low and high expression (fold change < 0.5 or > 2.0, respectively) is approximately 50%. For the cell to tissue comparison, all tissues show a statistically significant difference as compared to either the CHO-S or CHO DG44 cell lines. The proteins expressed at significantly higher levels in cells over tissues include proteins related to DNA replication, transcription, translation, and controlling cell apoptosis as expected to maintain rapid cell growth in exponential culture. Among the most highly expressed proteins in CHO-S and CHO DG44 are DNA-directed RNA polymerase II, eukaryotic translation initiation factor, histone H3.1t, general transcription factor 3C, 60S ribosomal protein, and apoptosis inhibitor 5.

In comparison to ovary tissue (Fig. 3E,I respectively, p < 0.01), CHO-S and CHO DG44 cells exhibited differences in expression patterns. This is somewhat surprising considering that CHO cells were derived from a mixture of ovary and the surrounding connective tissue. In Fig. 3E,I, the proteins colored blue and yellow represent those with higher expression in ovary or CHO cells, respectively. In addition to ovary, statistical differences in protein expression were observed for CHO-S and CHO DG44 cells and to lung tissue (Fig. 3D,H, p < 0.01). Similar to the comparison against ovary, an increase in upregulated proteins with higher expression was observed in the lung tissue in comparison to the cell lines. This suggests that tissue-specific functions may contribute to differences in expression patterns with cells regardless of the type of tissue.

Next, both CHO cell lines show statistically significant differences in outliers as compared against heart tissue (Fig. 3C,G, p < 0.01). Similar to the ovary and lung comparison, there are a greater number of proteins with higher expression in the heart tissue when compared to cell lines. Over 50% of the cells in the heart are cardiac fibroblasts, which contributes to the specificity of this organ^34^. In addition, the heart has endothelial, smooth muscle, and pacemaker cells. This high degree of specialization is likely influential for the differences in proteins between CHO and heart. Finally, both CHO-S and CHO DG44 also show a difference in expression when compared to the brain, which is also likely related to the high degree of specialization required for brain cells such as neurons and glia (Fig. 3B,F, p < 0.01).

Amongst the tissues, wide differential regulation, both upregulation and downregulation, can be observed when comparing tissues against each other (Fig. 3J–O). There is a greater difference in terms of the total percentage of outliers in the brain versus heart comparison (~ 62% total outliers for brain and ~ 51% total outliers for heart). Interestingly, only brain and heart tissues were found to have a statistically significant difference between each other (Table 3). All other tissue to tissue comparisons were found to have insignificant differences in the percentage of outliers. One reason for the relatively high number of outliers in brain tissue may be attributed to the distinct separation in terms of embryonic development from the other organs. The brain originates from the ectoderm whereas the circulatory system (heart), epithelial layer of the lungs, and ovary develop from the mesoderm^35^. Additional plots for the remaining comparisons (Supplementary Fig. 1) and a summary of the additional means comparison (Supplementary Table 2) are provided in the appendix.

**Figure 4:**
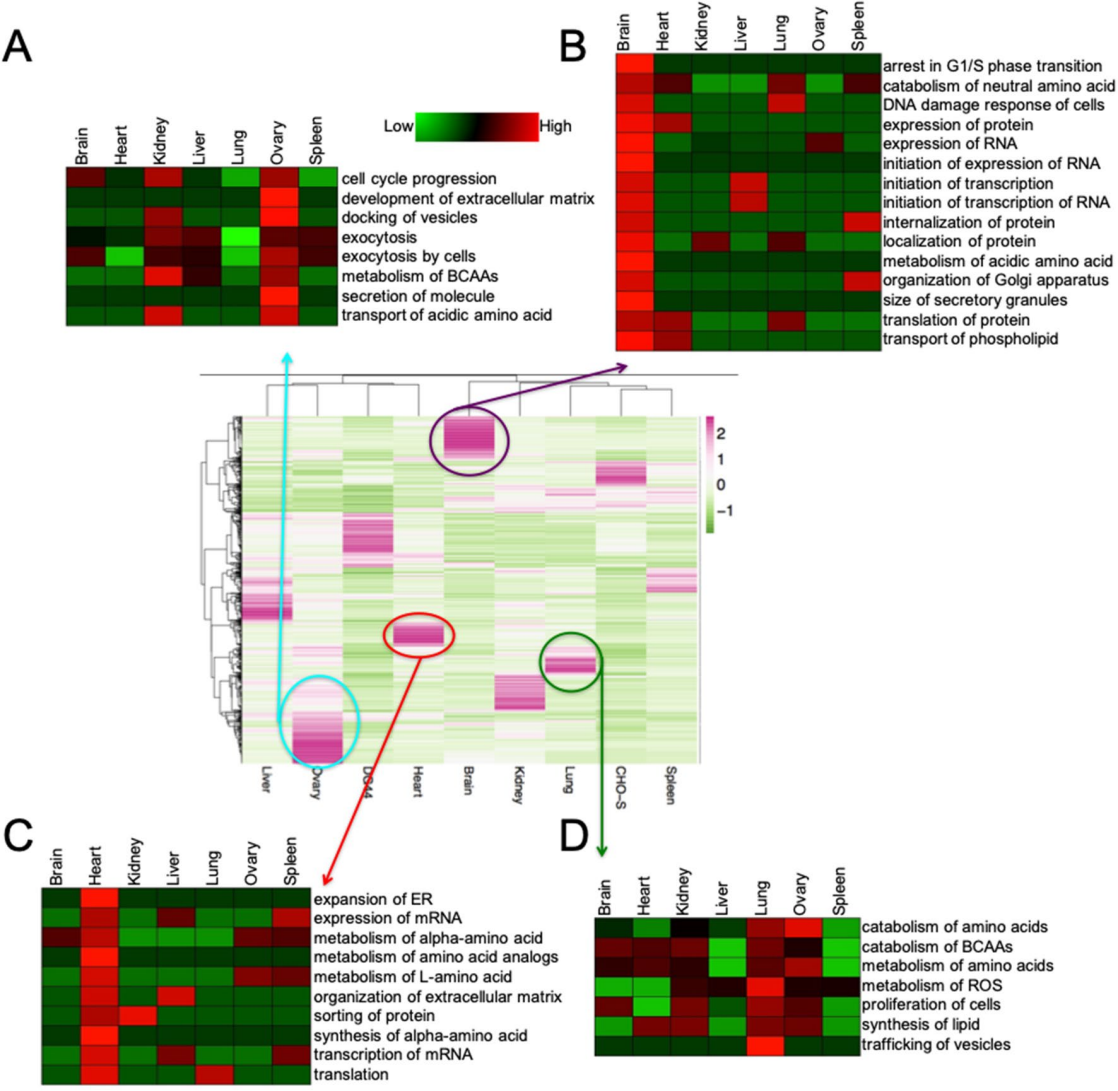
Center: heat map of highly expressed proteins. The top 200 proteins are plotted for each sample. Coloring is shown from low (green) to high (pink) abundance. Distinct clusters are shown for brain, lung, heart, and ovary tissues. From top to bottom: the protein functions exhibiting high expression for each tissue are shown relative to other tissues for ovary (**A**), brain (**B**), heart (**C**), and lung (**D**) plotted using Genesis software.

We also examined some of the top upregulated proteins in each tissue. Hierarchical clustering of protein expression is depicted in the center plot of Fig. 4 in which the color pink represents highly expressed proteins for each specific tissue. For each sample, the top 200 upregulated proteins, corresponding to approximately 3% of the total proteins in a tissue, were identified in order to highlight tissue specificity (Supplementary Table 3).

For example, disintegrin and metalloproteinase domain-containing proteins (ADAM7, 10, 15) were highly upregulated in hamster ovary (Supplementary Table 3). Indeed, ADAM 10 can control follicle formation by regulating the recruitment of ovarian follicle supporting cells^36^. Disintegrin would be useful for control of the extracellular matrix as agrees with results from the human ovary-specific proteome^37^. Similarly, highly upregulated brain proteins include amyloid beta A4 protein, neuron navigator 1 (NAV1), calcium and integrin binding protein, and serine/threonine protein kinase (Supplementary Table 3). NAV1 is specifically targeted to the nervous system^38^.

In contrast, the heart is a strong muscle that must contract continually and is predominantly composed of cardiomyocytes and fibroblasts. The human heart tissue proteome was found to have 201 upregulated proteins^37^, including retinol dehydrogenase (RDH1) which was also significantly upregulated in our hamster heart proteome (Supplementary Table 3). Previous studies have indicated that the knock-down of RDH1 led to abnormal neural crest cell migration and an abnormal heart loop in mutant embryos, signaling its importance in heart tissue^39^. We also observed high expression of proteins including pleckstrin homology domain-containing family F member 1-like, actin-related protein, glutathione S-transferase, protein O-glycosyltransferase, and Ras GTPase-activating protein (Supplementary Table 3).

We also identified the top 200 upregulated proteins in hamster lung tissue (Supplementary Table 3). Examples include branched chain aminotransferase, glycine *N*-methyltransferase, integrin alpha-6, and vesicle-associated membrane protein, indicating the importance in the lung of key metabolic and membrane process. As a comparison, the human proteome identified 183 genes highly expressed in the lung including similar membrane and secretory proteins^37^. Indeed, the lung is particularly adept at expressing membrane and secreted proteins such as surfactants and solute carrier proteins^40^. Analysis of the most abundant proteins for each tissue or cell line can, at least in some cases, relate to key function and roles of specific tissues and provide targets of opportunity for genetic engineering of CHO cell lines.

### IPA pathway analyses

Next, the proteins were annotated with gene symbols in order to perform pathway analysis. Proteins with fold change values of less than 0.5 or greater than 2.0 were used for each comparison in order to determine downregulation and upregulation of pathways in the IPA software. As shown in Fig. 4, protein intensity hierarchical clustering was used to identify proteins with significantly higher expression in a specific tissue.

Protein functions identified in IPA that are enriched in the brain include G1/S phase arrest, metabolism of acidic amino acids, and organization of the Golgi apparatus, and transport of phospholipids (Fig. 4B). The Golgi plays a central role in cholesterol and other lipid metabolism; almost 25% of the human body’s unesterified cholesterol is present in the brain^41^. Additionally, glycosphingolipids are abundant in the nervous system, and are synthesized in the endoplasmic reticulum and completed in the Golgi apparatus^42^. Acidic amino acids, including glutamate and aspartate, serve important signaling functions in the brain, so an increase in their metabolic activity would be expected, especially since these metabolites are not readily obtained from the diet^43,44^.

Pathways enriched in the lung include catabolism of branched chain amino acids (BCAAs), metabolism of reactive oxygen species (ROS), trafficking of vesicles, and synthesis of lipids (Fig. 4D). The lung shows high secretory capacity and thus trafficking of the secretory products through vesicles is important^45^. Furthermore, the lung is the source of surfactants, composed of 90% lipids; thus, lipid synthesis will be an important component of its function^46,47^. The lungs are also particularly sensitive to hypoxia occurring at high altitude. The lung responds to these hypoxic conditions through signaling, including the release of ROS species to trigger hypoxia-inducible factor (HIF)^48^. Thus, the capacity for ROS metabolism may be critical for lung function and adaptation to changes in oxygen levels in different environments.

Similarly, in the heart, enriched pathways include metabolism of alpha-amino acid, sorting of protein, and translation (Fig. 4C). Amino acid metabolism was studied in rat heart with amino acid levels rising up to fivefold higher than plasma levels^49^, accompanied by increases in ribosome activity and translation^50^. Finally, protein functions enriched in the ovary include development of extracellular matrix, docking of vesicles, and exocytosis (Fig. 4A). The matrix of ovary tissue is important for numerous physiological activities, including growth, migration, and differentiation, and the composition changes during ovulation^31^, critical to the fertility process^51^. Further, malfunctions of the matrix are observed during ovarian cancer^52^. High levels of extracellular matrix components in Chinese hamster ovaries are in agreement with our previous research examining the hamster ovary tissue proteome using label-free proteomics approaches^12^.

### GO functional analysis

For GO functional analysis, gene symbols were annotated for biological processes in order to group proteins with similar biological relevance. The biological process GO category was analyzed to determine enrichment and depletion p-values for the cell-to-tissue and tissue-to-tissue comparisons. The appendix lists the top 10 most enriched biological processes for CHO-S (Supplementary Table 4) and CHO DG44 (Supplementary Table 5) comparisons with tissue. Enrichment is determined by hypergeometric distribution, with a p-value of < 0.05 used for significance.

Biological processes, representing a biological function involving the gene product, complements the pathway analysis in “IPA pathway analyses” shown above^27^. In both CHO-S and CHO DG44, the most common biological processes enriched involve DNA and mRNA processing, and metabolism. Some of the most common enriched processes in CHO-S include mRNA processing and splicing, DNA replication and repair, transcription, cell cycle, chromatin modification, and chromosome condensation.

In comparison, signaling, transport, and adhesion were significantly enriched across the different hamster tissues. Enriched brain biological processes relative to the CHO cell lines include ion transport, axon guidance, synaptic transmission, and metabolic process, among others^27^. Ion transport helps to maintain the stability of cerebral function due to the key roles that ions play in currents and synaptic transmission^53^. Abnormal distributions of ions in the brain can lead to defects in neuronal function including seizures and depression.

Enriched biological processes in the heart compared to the CHO cell lines show functions related to circulation and heart function such as vasoconstriction, sodium-independent organic ion transport, complement activation, blood coagulation, and lipoprotein metabolism. For example, complement activation involves mannose-binding lectin and complement components, C3, C5, and CD59. The complement cascade can be activated during heart disease or failure, especially in cases of myocardial ischemia and reperfusion^54,55^. Biological processes that are enriched in the lung as compared to the CHO cell lines include G-protein coupled receptor signaling pathway, innate immune response, vesicle-mediated and transmembrane transport, and signal transduction. Indeed, vesicle transport is an important component of secretory pathway machinery. Unraveling the complexities of lung secretions may yield new insights into ways by which secretion differs in tissues and cell lines. Signaling through G-protein coupled receptors and other pathways is an important proinflammatory response in lungs, which can undergo modifications leading to lung cancers^56,57^. Not surprisingly, protection of the lungs using the host innate immune response is critical as this organ is exposed to a variety of pathogens including bacterial, fungal, and viral, during breathing^58,59^.

Finally, enriched biological processes in the ovary highlight differences between the cells and tissue around the region from which CHO cells were derived. Enriched ovarian biological processes include transmembrane transport, protein transport, vesicle-mediated transport, cell adhesion, and cell death. When vesicle production rate was quantified in the ovary, the turnover indicated high vesicle recycling across the endomembrane system^59^, which is consistent with these proteomic results. Cell adhesion is also important to ovary function and follicle maturation through interactions with the extracellular matrix and direct cell–cell contacts. Mutation of the ovarian surface is causative of approximately 90% of malignant ovarian tumors^60,61^.

## Concluding remarks

The results from the Chinese hamster proteome provide new insights into global protein expression across a wide variety of tissues and multiple cell lines. These differences highlight the role of tissues in executing key organ functions which require a specific metabolic processes, such as transport and communication, in comparison to CHO cells, which are focused on replication and gene expression, characteristics useful for rapid growth and the production of biologics. Because of their relevance to biomedicine and the biotechnology industry, we compared the tissue proteome to CHO cell lines and each other in order to identify functional differences in expression across tissues and cell lines. Specifically, we observed enrichment of many physiological pathways in tissues that were not enriched in cells, such as ion, protein, and vesicle transport, signal transduction, and cell adhesion. Often, these differences correlated with specific tissue functions while the activities in cell lines were often correlated with DNA replication, cell cycle, or RNA processing. Furthermore, some of the proteins with high expression in lung, ovary, or other tissues versus CHO, such as vesicle-mediated and protein transport, provide significant opportunities for CHO cell engineering going forward. In this way, the study expands on our CHO and Chinese hamster tissue knowledge base by virtue of establishing an atlas to differentiate proteins across cells and tissue for this critically important biotechnological and biomedical host species. Indeed, this comparison has enabled us to appreciate the changing proteomic landscape across cells and tissues and furthermore to recognize how the expressed proteins from different cell types can represent signatures for some of their key physiological or biotechnological functions.

## Supplementary information

Supplementary Information 1.

Supplementary Information 2.

## Data Availability

The complete list of identified proteins and fold change ratios are listed in Supplementary Table 1 and are searchable online through the NIST database (https://peptide.nist.gov).
