# Supplementary material for: Expanded Chinese hamster organ and cell line proteomics profiling reveals tissue-specific functionalities
Source: Sci Rep. 2020 Sep 28;10:15841. doi: 10.1038/s41598-020-72959-8 (PMC7522264; doi:10.1038/s41598-020-72959-8)
Supplement: Supplementary file 1 — Supplementary Information 1. [file 41598_2020_72959_MOESM1_ESM.docx]

**Expanded Chinese Hamster Organ and Cell Line Proteomics Profiling Reveals Tissue-Specific Functionalities**

Kelley Heffner^1,2^, Deniz Baycin Hizal^1^, Natalia I. Majewska^1,2^, Swetha Kumar^1^, Venkata Gayatri Dhara^1^, Jie Zhu^2^, Michael Bowen^3^, Diane Hatton^2^, George Yerganian^4,6^, Athena Yerganian^4^, Robert O’Meally^5^, Robert Cole^5^, Michael Betenbaugh*^1^

^1^Johns Hopkins University, Department of Chemical and Biomolecular Engineering, Baltimore, MD, USA

^2^AstraZeneca, Cell Culture and Fermentation Sciences, Gaithersburg, MD, USA

^3^Allogene Therapeutics, Product and Process Development, South San Francisco, CA, USA

^4^Cytogen Research and Development, Inc, Boston, MA, USA

^5^Johns Hopkins School of Medicine, Department of Pathology, Baltimore, MD, USA

^6^Deceased

*Correspondence to: beten@jhu.edu

**Appendix**

Supplementary Table 1a. TMT1 protein names and fold changes. A list of the protein accession numbers, protein descriptions, and metrics for each of the samples included in experiment 1. Channels are expressed as fold change, using CHO-S technical replicate as basis for control in columns H, J, L, N, P, R, T, V, and X. (See Excel file)

Supplementary Table 1b. TMT2 protein names and fold changes. A list of the protein accession numbers, protein descriptions, and metrics for each of the samples included in experiment 2. Channels are expressed as fold change, using CHO-S technical replicate as basis for control in columns H, J, L, N, P, R, T, V, and X. (See Excel file)

Supplementary Table 2. Means Comparison using Student’s t-test for Total Percentage of Outliers

| p-value | **CHO-S** | **CHO DG44** | **Brain** | **Heart** | **Lung** | **Ovary** | **Kidney** | **Liver** | **Spleen** |
| --- | --- | --- | --- | --- | --- | --- | --- | --- | --- |
| **CHO-S** | n/a | 0.067 | <0.01* | <0.01* | <0.01* | <0.01* | <0.01* | <0.01* | <0.01* |
| **CHO DG44** | 0.067 | n/a | <0.01* | <0.01* | <0.01* | <0.01* | <0.01* | <0.01* | <0.01* |
| **Brain** | <0.01* | <0.01* | n/a | <0.01* | 0.10 | 0.12 | 0.12 | 0.054 | <0.01* |
| **Heart** | <0.01* | <0.01* | <0.01* | n/a | 0.11 | 0.051 | 0.087 | 0.19 | 0.61 |
| **Lung** | <0.01* | <0.01* | 0.10 | 0.11 | n/a | 0.79 | 0.90 | 0.72 | 0.043* |
| **Ovary** | <0.01* | <0.01* | 0.12 | 0.051 | 0.79 | n/a | 0.89 | 0.51 | 0.018* |
| **Kidney** | <0.01* | <0.01* | 0.12 | 0.087 | 0.90 | 0.89 | n/a | 0.63 | 0.035* |
| **Liver** | <0.01* | <0.01* | 0.054 | 0.19 | 0.72 | 0.51 | 0.63 | n/a | 0.082 |
| **Spleen** | <0.01* | <0.01* | <0.01* | 0.61 | 0.043* | 0.018* | 0.035* | 0.082 | n/a |

*p-value < 0.05 indicates that the outlier comparison is statistically significant.

Supplementary Table 3. Top 200 proteins. A list of the accession numbers and fold change for the most highly expressed proteins in each sample, using CHO-S technical replicate as basis for control. (See Excel file)

Supplementary Table 4. Top 10 most enriched biological processes for CHO-S and tissues

| **Comparison** | **Ranking** | **Enriched in CHO-S** | **Enriched in Hamster Tissue** |
| --- | --- | --- | --- |
| CHO-S vs. Brain | 1 | Transcription, DNA-templated | Transport |
|  | 2 | Regulation of transcription, DNA-templated | Signal transduction |
|  | 3 | Gene expression | Metabolic process |
|  | 4 | Positive regulation of transcription from RNA polymerase II promoter | Small molecule metabolic process |
|  | 5 | DNA repair | Ion transport |
|  | 6 | Transcription from RNA polymerase II promoter | Synaptic transmission |
|  | 7 | RNA metabolic process | Oxidation-reduction process |
|  | 8 | Viral process | Transmembrane transport |
|  | 9 | Cellular protein metabolic process | Cell adhesion |
|  | 10 | mRNA processing | Axon guidance |
| CHO-S vs. Heart | 1 | DNA replication initiation | Brown fat cell differentiation |
|  | 2 | DNA strand elongation involved in DNA replication | Retinoid metabolic process |
|  | 3 | DNA replication | Lipoprotein metabolic process |
|  | 4 | DNA unwinding involved in DNA replication | Positive regulation of vasoconstriction |
|  | 5 | Cell cycle | Cartilage development involved in endochondral bone morphogenesis |
|  | 6 | DNA recombinase assembly | Regulation of blood coagulation |
|  | 7 | Chromosome condensation | Complement activation |
|  | 8 | Nucleotide-excision repair, DNA damage removal | Protein heterotrimerization |
|  | 9 | Nucleotide-excision repair, DNA gap filling | Cilium assembly |
|  | 10 | Telomere maintenance via recombination | Prostaglandin metabolic process |
| CHO-S vs. Lung | 1 | Chromatin modification | G-protein coupled receptor signaling pathway |
|  | 2 | mRNA processing | Vesicle-mediated transport |
|  | 3 | RNA splicing | Innate immune response |
|  | 4 | DNA repair | Intracellular signal transduction |
|  | 5 | Cellular protein metabolic process | Cell adhesion |
|  | 6 | Translation | Phosphorylation |
|  | 7 | RNA metabolic process | Transmembrane transport |
|  | 8 | Transcription, DNA-templated | Signal transduction |
|  | 9 | Translational initiation | Proteolysis |
|  | 10 | Transcription from RNA polymerase II promoter | Blood coagulation |
| CHO-S vs. Ovary | 1 | Cellular response to DNA damage stimulus | Synaptic transmission |
|  | 2 | Translational initiation | Blood coagulation |
|  | 3 | Ubiquitin-dependent protein catabolic process | Transmembrane transport |
|  | 4 | mRNA splicing, via spliceosome | Cell adhesion |
|  | 5 | Cell division | Signal transduction |
|  | 6 | Cell cycle | Metabolic process |
|  | 7 | Intracellular protein transport | Small molecule metabolic process |
|  | 8 | Transcription from RNA polymerase II promoter | Transport |
|  | 9 | Gene expression | Protein transport |
|  | 10 | RNA metabolic process | Vesicle-mediated transport |

Supplementary Table 5. Top 10 most enriched biological processes for CHO DG44 and tissues

| **Comparison** | **Ranking** | **Enriched in CHO DG44** | **Enriched in Hamster Tissue** |
| --- | --- | --- | --- |
| CHO DG44 vs. Brain | 1 | Transcription, DNA-templated | Transport |
|  | 2 | Regulation of transcription, DNA-templated | Signal transduction |
|  | 3 | Gene expression | Metabolic process |
|  | 4 | Transcription from RNA polymerase II promoter | Cell adhesion |
|  | 5 | RNA splicing | Phosphorylation |
|  | 6 | DNA repair | Intracellular signal transduction |
|  | 7 | mRNA processing | Ion transport |
|  | 8 | RNA metabolic process | Synaptic transmission |
|  | 9 | Cellular protein metabolic process | Small molecule metabolic process |
|  | 10 | Viral process | Axon guidance |
| CHO DG44 vs. Heart | 1 | Mitotic nuclear division | Cilium assembly |
|  | 2 | DNA replication | Cilium morphogenesis |
|  | 3 | DNA replication initiation | Complement activation |
|  | 4 | Mitotic cell cycle | Protein heterotrimerization |
|  | 5 | rRNA processing | Positive regulation of smoothened signaling pathway |
|  | 6 | DNA strand elongation involved in DNA replication | Phospholipid efflux |
|  | 7 | Cell cycle | Cell-substrate junction assembly |
|  | 8 | Chromosome segregation | Chylomicron remnant clearance |
|  | 9 | Translesion synthesis | Sodium-independent organic anion transport |
|  | 10 | Mitotic metaphase plate congression | Cellular response to stress |
| CHO DG44 vs. Lung | 1 | Ubiquitin-dependent protein catabolic process | Membrane organization |
|  | 2 | rRNA processing | Vesicle-mediated transport |
|  | 3 | Viral life cycle | G-protein coupled receptor signaling pathway |
|  | 4 | Chromatin modification | Regulation of small GTPase mediated signal transduction |
|  | 5 | mRNA processing | Axon guidance |
|  | 6 | RNA splicing | Ion transmembrane transport |
|  | 7 | DNA repair | Small GTPase mediated signal transduction |
|  | 8 | Cellular protein metabolic process | Ion transport |
|  | 9 | Translation | Intracellular signal transduction |
|  | 10 | RNA metabolic process | Single organismal cell-cell adhesion |
| CHO DG44 vs. Ovary | 1 | Mitotic nuclear division | Cell death |
|  | 2 | Cell division | Vesicle-mediated transport |
|  | 3 | Cellular response to DNA damage stimulus | Synaptic transmission |
|  | 4 | Mitotic cell cycle | Protein transport |
|  | 5 | DNA repair | Nervous system development |
|  | 6 | Transcription from RNA polymerase II promoter | Proteolysis |
|  | 7 | mRNA processing | Transmembrane transport |
|  | 8 | RNA splicing | Cell adhesion |
|  | 9 | Regulation of transcription from RNA polymerase II promoter | Intracellular signal transduction |
|  | 10 | Cell differentiation | Transport |

Supplementary Figure 1. Protein Intensity Additional Comparisons. Overall distribution of protein intensity in hamster tissues. Protein intensity plotted as log_2_ transformed protein intensity of biological replicates. Yellow represents proteins highly expressed for y-axis. Blue represents proteins highly expressed for x-axis.
